# Translationally Controlled Tumour Protein (TCTP) is present in human cornea and increases in herpetic keratitis

**DOI:** 10.1186/1746-1596-7-90

**Published:** 2012-08-01

**Authors:** Cinzia Batisti, Maria R Ambrosio, Bruno J Rocca, Gian M Tosi, Jean C Sanchez, Felice Arcuri, Marcella Cintorino, Sergio A Tripodi

**Affiliations:** 1 Departments of Ophthalmology, University of Siena, Siena, Italy; 2 Department of Human Pathology and Oncology, Anatomical Pathology Section, University of Siena, via delle Scotte 6, 53100, Siena, Italy; 3 Geneva Proteomics Center, Central Clinical Chemistry Laboratory, Geneva University Hospital, Geneva, Switzerland

## Abstract

**Background:**

Translationally Controlled Tumour protein is a multifunctional calcium binding protein which has an important role in apoptosis, calcium levels balance and immunological response. The aim of this study was to evaluated the presence and distribution of TCTP in healthy human corneas and to identify and characterize the presence and distribution of this protein in human normal cornea. Since recent studies suggest that apoptosis, calcium levels and immunological mechanisms play a role in the pathogenesis of herpetic stromal keratitis, we studied TCTP expression in this disease.

**Methods:**

We evaluated the expression of TCTP at both RNA messanger and protein level by using reverse transcriptase analysis, immunoblotting and immunohistochemistry in 10 healthy samples cornea: four obtained after penetrating keratoplasty and six from eyes enucleated for other pathologies. Finally, we analysed by immunohistochemistry ten paraffin-embedded samples of Herpes simplex virus keratitis collected at Siena Department of Human Pathology and Oncology: 5 had clinically quiescent disease and 5 had active corneal inflammation.

**Results:**

Reverse transcriptase and immunoblotting demonstrated TCTP expression in cornea as a 22,000 Da molecular weight band corresponding to the molecular weight of this protein. Immunohistochemically, all the layers of normal corneal epithelium showed TCTP cytoplasmic expression. TCTP was, also, observed in keratocytes and in the endothelium. In Herpes simplex virus keratitis samples, strong expression of TCTP was evident in stromal cells, in the inflammatory infiltrate and in neo-vessels.

**Conclusions:**

In this preliminary study we demonstrated, for the first time, the presence of TCTP in human cornea, suggesting a potential role in the pathogenesis of herpes virus keratitis.

**Virtual Slides:**

The virtual slide(s) for this article can be found here: http://www.diagnosticpathology.diagnomx.eu/vs/3306813447428149

## Background

Translationally controlled tumour protein (TCTP), also known as fortilin [1] or TPT1 [2] is a ubiquitously expressed protein of 21 kDa in mice and 23 kDa in humans, expressed in all eukaryotes. It bears no sequence similarity with any other known protein [1]. It is encoded by a gene that maps to chromosome 13q14.13 [1,3] and its expression is highly regulated at transcriptional and translational level and by a wide range of extracellular signals. Its name originates from the observation that TCTP transcripts accumulate in resting cells and are rapidly translated into the protein when the cells require it [4]. Although TCTP was first considered a tumour protein [5], its expression is not limited to cancer, as it has been found in normal cells and tissues [6]. Since its discovery by Yenofsky [7], it has become clear that TCTP is a multifaceted protein, implicated in many biological processes and exerting biological activity at extracellular and intracellular level [4]. TCTP participates in cell growth, cell cycle progression, division and proliferation [8]. An anti-apoptotic function of TCTP in human cancer cells has also been identified. This function may be related to calcium binding [9-12] and inhibition of Bax dimerization [4]. The interaction between TCTP and p53 prevents apoptosis by destabilizing p53 [13]. TCTP is also considered a heat shock protein with chaperone-like activity [14]. It functions as an IgE-dependent histamine releasing factor, having cytokine-like activity in acute allergic response and being involved in immunological response [15]. Association with a cytoskeletal component, F-actin, and a role in cell shape regulation were also recently discovered [16], as well as its capacity to bind tubulin and serve as a substrate for Polo-like kinase 1 (Plk-1) [4].

Ocular vision depends on corneal transparency and shape. These properties depend on the balance between cell proliferation and apoptosis, both mechanisms regulated by TCTP [17]. The presence and distribution of TCTP in the human cornea has not yet been fully analysed. Studies in the literature have used proteomic techniques and been limited to cultured keratocytes [18]. Here we evaluated the presence and distribution of TCTP in healthy human corneas for the first time by immunoblotting, reverse transcriptase analysis and immunohistochemistry. Since recent studies suggest that apoptosis [19], calcium levels [20] and immunological mechanisms [21] play a role in the pathogenesis of herpetic stromal keratitis (HSK), we studied TCTP expression in this disease.

## Materials and methods

### Ethics Statement

Ethics approval for this study was obtained from the Institutional Review Board at the University of Siena (Italy). Informed written consent was obtained in all cases.

### Patients

We used 10 samples of healthy corneal tissue, four obtained after penetrating keratoplasty (PKP) and six from eyes enucleated for retinoblastoma, in which the anterior segment was tumor-free. Ten other corneal tissue samples were collected from patients undergoing PKP for sequelae of HSK. The diagnosis of HSK was based on clinical and slit lamp findings. PKPs were performed consecutively from August 2010 to December 2011.

### Tissue preparation

**Figure 1:**
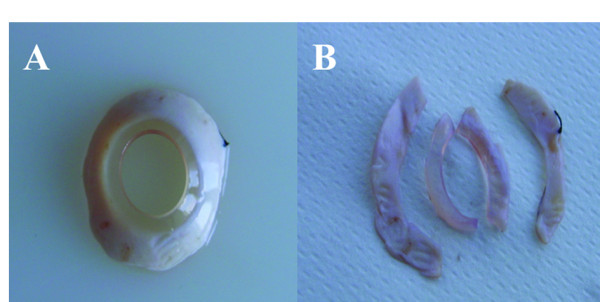
**A-B. The surgical specimen. **Peripheral corneal tissue obtained after penetrating keratoplasty.

The surgical specimens obtained after PKP were grossly examined and halved (Figure 1A-B). Half of the samples were rinsed in sterile HBSS (Hanks balanced salt solution) at room temperature, blotted dry and dissected with a razor blade. To analyse protein and RNA, they were snap-frozen and stored in liquid nitrogen. The other half were fixed in 10% buffered formalin and embedded in paraffin. From paraffin-embedded blocks of PKP and enucleation samples, 4-μm-thick sections were obtained and processed for histology and immunohistochemistry. Anti-human TCTP mouse monoclonal antibody was prepared as previously described [22].

### Western blot analysis

Frozen corneal tissue samples were thawed, reduced to small pieces with a razor blade and homogenized on ice with a Polytron blender (Kinematica, Lucerne, Switzerland) in lysis buffer [50 mMTris–HCl, 5 mM magnesium acetate, 0.2 mM EDTA, 0.5 mMdithiothreitol, 10% (vol/vol) glycerol, 0.2% (vol/vol) Triton X-100 (pH 7.5)] supplemented with a protease-inhibitor cocktail (Sigma Chemical Co, Milan-Italy), three times for 20 seconds each. Tissues were centrifuged at 750 x *g* for 10 min at 4°C and the supernatant assayed for total protein content and stored at −80°C. Western blot analysis was carried out as previously described [23].

### Detection of TCTP mRNA

Total RNA was extracted using the method of Chomczynski and Sacchi. Frozen tissues were crushed with a pestle and homogenized in Tri-Reagent (Life Technologies, Monza-Italy) with a Polytron blender (Kinematica, Luzern-Switzerland). Cell pellets were thawed and immediately lysed with Tri-Reagent. RNA extraction was carried out as recommended by the manufacturer. TCTP mRNA was detected by reverse transcriptase-polymerase chain reaction (RT-PCR) as previously described [23]. The products were separated by 2.0% agarose gel electrophoresis, visualized by ethidium bromide staining, and photographed.

### Immunohistochemical analysis

Immunohistochemistry was performed using the alkaline phosphatase–anti-alkaline-phosphatase (APAAP) method. Sections were dewaxed, rehydrated and washed in Tris-buffered saline [TBS, 20 mMTris–HCl, 150 mMNaCl (pH 7.6)]. Antigen retrieval was carried out by incubating sections in sodium citrate buffer (10 mM, pH 6.0) in a microwave oven at 750 W for 5 min. Sections were incubated with an anti-human TCTP monoclonal antibody diluted 1:100 in TBS overnight at 4°C. Slides were washed and incubated for 30 min with a rabbit anti-mouse antibody (Dako, Milan, Italy, dilution 1:30) and finally incubated for 30 min with APAAP complex (Dako, dilution 1:50) in TBS. The alkaline phosphatase reaction was detected using fuchsin and naphthol. TCTP expression was evaluated semiquantitatively by HSCORE, as previously described [24]. Placental tissue served as external positive control [10].

### Statistical analysis

Statistical analysis was performed using a statistical software package (SYSTAT-7). Differences in TCTP pattern between cases grouped according to inflammation and neovascularization (groups 1 and 2) were analysed using the Chi-square test and Fisher’s exact test. Statistical significance was set at *P* ≤ 0.05.

## Results

### Normal cornea

#### RT-PCR analysis of TCTP mRNA levels

**Figure 2:**
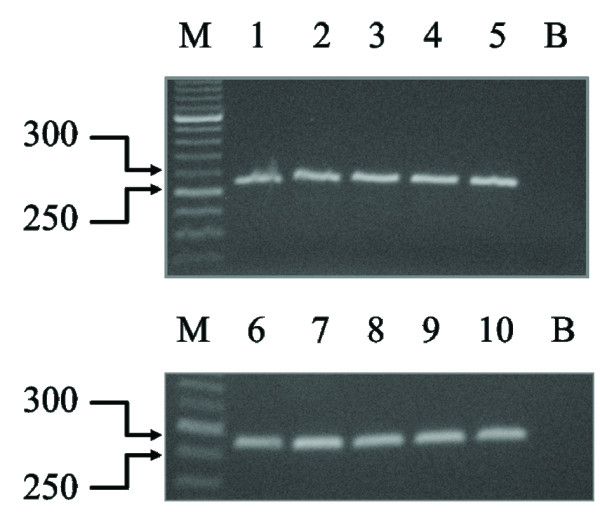
**RT-PCR analysis of TCTP mRNA levels in the 10 samples. **One microgram of total RNA was reverse transcribed and amplified in the presence of TCTP primers. Thirty cycles were run for both PCRs. The size of the MW markers is indicated. B, blank.

To evaluate steady-state levels of TCTP mRNA, total RNA extracted from tissues was examined by RT-PCR. When amplification was carried out in the presence of human TCTP primers, an intense band corresponding to the TCTP product was obtained on the agarose gel from the cDNA of each corneal tissue specimen (Figure 2).

#### Western blot analysis

**Figure 3:**
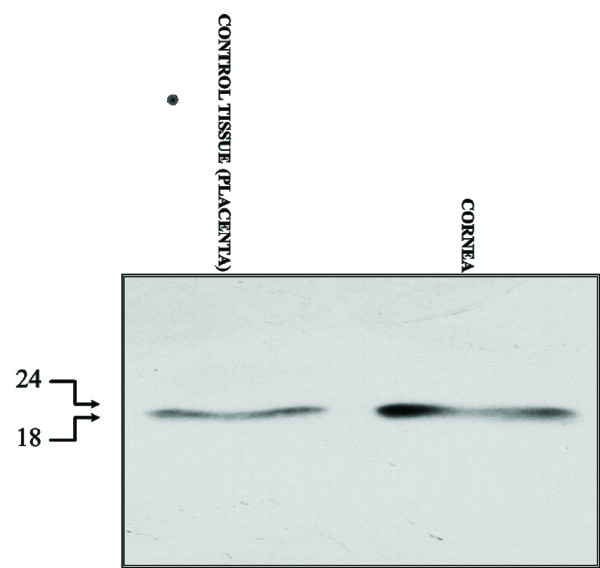
**Western blot profile. **Twenty micrograms of total protein were loaded onto 12% polyacrylamide gel, fractionated, blotted onto nitrocellulose and exposed overnight to an anti-TCTP antibody. The position of MW markers, in kDa, is indicated. Control tissue was placenta.

To further confirm the presence of TCTP in our specimens, Western blot analysis was carried out. After fractionation on SDS–PAGE, proteins of all specimen homogenates were blotted onto nitrocellulose and the filter exposed to an anti-TCTP antibody. A single band of the approximate molecular weight (MW) of 22,000 Da was detected in all specimens (Figure 3). Staining was abolished by probing blots identical to those described above with the antibody pre-absorbed with recombinant TCTP. Control tissue was represented by placenta, as previously described [10].

#### Immunohistochemistry

**Figure 4:**
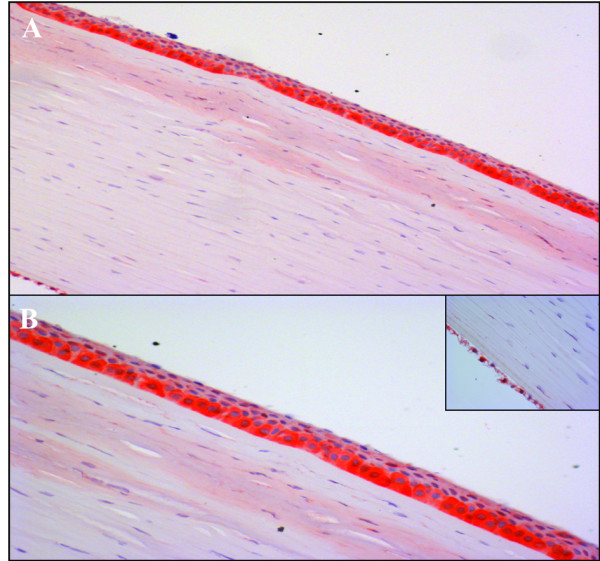
**Immunohistochemical profile of healthy corneal tissue. **Positive TCTP staining was observed in the basal and intermediate cell layers of the corneal epithelium (A-B); weak and focal staining was observed in keratocytes and along the endothelial cell layer (B, inset) [A, original magnification (O.M) 2.5x; B, O.M. 5x; inset, O.M. 2.5x].

Immunohistochemical analysis performed with the anti-TCTP antibody showed cytoplasmic staining. TCTP expression was observed in the basal (Figure 4A) and intermediate (Figure 4B) cell layers of corneal epithelium with HSCOREs of 2 and 1, respectively. TCTP was also evident in keratocytes and endothelial cells with a HSCORE of 1 (Figure 4B, inset).

### HSK

**Figure 5:**
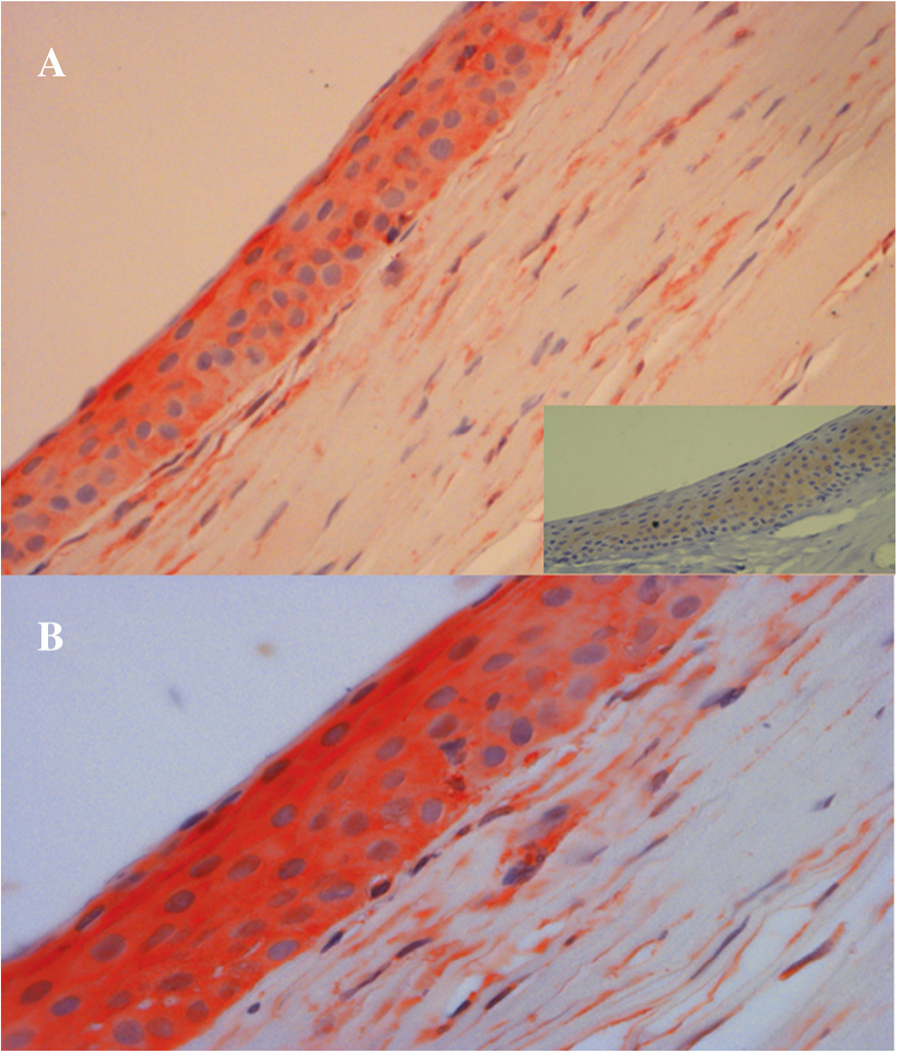
**Immunohistochemical profile of corneal tissue with HSK. **In active keratitis, strong TCTP staining was observed throughout all layers of the corneal epithelium, as well as in stromal cells (A-B), whereas in quiescent disease little or no expression was present (A, inset) [A, O.M 10x; B, O.M. 20x; inset, O.M. 20x].

Of the 10 specimens from patients with HSK, 5 had active keratitis (moderate to severe inflammation and neovascularization) and 5 had quiescent disease (absent or mild inflammation). In the active HSK samples, intense and diffuse (HSCORE 3) TCTP staining was observed in stromal cells throughout all layers of the corneal epithelium (Figure 5A-B). In the quiescent disease samples, TCTP expression was weak (HSCORE 1), present only in corneal epithelium with little or no immunohistochemical expression in the stroma (Figure 5A, inset) (p = 0.001).

## Discussion

TCTP is a highly conserved, ubiquitously expressed protein involved in multiple biological activities, including apoptosis [13]. An extensive review of the literature showed that previous studies on TCTP in the cornea used proteomics techniques were limited to cultured keratocytes [18]. In the present study we demonstrated the presence of TCTP in human corneal tissue by RT-PCR and immunoblotting, and confirmed these findings by immunohistochemistry using an antibody against TCTP. Since apoptosis plays a key role in HSK and is controlled by TCTP, we also attempted to find a pathogenic correlation between TCTP and HSK, evaluating TCTP expression in corneal samples with HSK. TCTP inhibited the proapoptotic proteins Bax and BCLxL, promoters of membrane attack complex (MAC) pore formation via dimerization of the mitochondrial membrane. TCTP inserts itself in the mitochondrial membrane, preventing Bax from dimerizing. This inhibits MAC pore formation and prevents any flux of apoptosis promoting factors into the cell. TCTP also binds to Mcl-1, stabilizing it and enhancing its anti-apoptotic activity. In addition, TCTP was shown to bind p53 and prevent apoptosis by destabilizing the protein and repressing transcription of p53 [4]. A correlation was found between the degree of inflammation and expression of TCTP in corneal epithelium, as well as in inflammatory infiltrate in HSK patients with active disease [25]. In the case of mild inflammation, we found low expression of TCTP, limited to neovessels and keratinocytes. These results suggest a double role of TCTP in herpes keratitis: as an antiapoptotic protein in corneal cells and as a cytokine-like protein in the stroma, where TCTP may be secreted by inflammatory cells, probably monocytes, in the extracellular milieu. Apoptosis has been shown to limit viral invasion: corneal infection with wild-type virus results in apoptosis of a number of infected epithelial cells [26]. Primary herpes simplex virus-1 (HSV-1) corneal infection stimulates apoptosis of anterior keratocytes in the stroma underlying the site of epithelial injury. TCTP may modulate the keratocyte apoptotic response, limiting stromal damage as well as triggering the keratocyte apoptotic response via soluble mediators released by epithelial injury secondary to corneal HSV-1 infection. Considering the importance of apoptosis as a general mechanism for eliminating normal and pathologically altered cells, manipulation of apoptosis may offer new possibilities for treating HSK.

We also found a huge amount of TCTP in the extracellular stroma of actively inflamed HSK, which is in line with previous studies on broncho-alveolar lavage fluids of patients with eosinophilic pneumonia and asthma [27,28]. A mechanism by which extracellular TCTP might intervene through cytokine-like activity in the complex pathogenesis of HSK is by regulating the immune-mediated response to herpes simplex virus. In fact, HSK is currently thought to be an immune-mediated disease. CD4 positive Th1 cells are involved in the development of inflammatory HSK corneal lesions: infiltration of CD4 positive Th1 cells into the cornea causes release of cytokines, mainly interleukin (IL)-2 and interferon (IFN)-γ, which elicit an inflammatory response and recruit neutrophils and macrophages. TCTP is known to inhibit the release of IL-2 in human peripheral blood T cells and Jurkat T cells [29] and a role of TCTP in the modulation of T regs apoptosis/survival was recently demonstrated [30].

Repair of damage after each infection progressively impairs the biomechanical properties of the cornea. The repair process or wound healing of corneal epithelium is complex, including cell migration, proliferation and differentiation, all of which depend on calcium-mediated signals [31]. Calcium concentrations vary during wound healing, thus inducing variations in cell motility, morphology and adhesion that are all essential to the process [32]. Calcium levels are regulated by TCTP [6].

## Conclusions

In this study we demonstrated the presence and distribution of TCTP in human corneal tissue and its expression in HSK, probably related to inflammation and neovascularization. These findings suggest a multifunctional role of TCTP in corneal wound healing, HSV-1 corneal infection, IgE-mediated hypersensitivity reactions and maintenance of corneal transparency and shape [16] by interaction with cytoskeletal proteins. Further *in vivo* and *in vitro* studies are needed to fully understand the pathophysiological roles of TCTP in corneal diseases, considering that only 20 samples were tested for TCTP and that no data on its role in HSK and other types of keratitis are available in the literature. Since a series of pharmaceutical compounds decrease TCTP expression by down-regulation of its expression at protein level, broader studies may help to determine whether TCTP may act as a target for drugs, contributing to new alternatives for the treatment of HSK, avoidance of complications and reduction of relapses [33].

## Abbreviations

TCTP: Translationally controlled tumour protein; PLK1: Polo-like kinase 1; HSK: Herpes stromal keratitis; PKP: Penetrating keratoplasty; HBSS: Hanks balanced salt solution; RT-PCR: Reverse transcriptase-polymerase chain reaction; APAAP: Alkaline phosphatase–anti-alkaline-phosphatase; TBS: Tris-buffered saline; MAC: Membrane attack complex; HSV-1: Herpes simplex virus-1; IL: Interleukin; IFN: Interferon; MW: Molecular weight.

## Competing interest

The authors declare that they have no competing interests.

## Authors’ contributions

CB and MRA wrote the paper; BJR analysed the histological and immunohistochemical sections; FA conducted the molecular biology experiments; GMT acquired clinical data; JCS contributed reagents; MC contributed his expertise and fruitful discussion; SAT coordinated the work and gave final approval of the version to be published. All authors read and approved the final manuscript.
